# The clinicopathologic features of uterine inflammatory myofibroblastic tumor: A case report

**DOI:** 10.1097/MD.0000000000041310

**Published:** 2025-01-17

**Authors:** Xiaoming Pan, Jian Zou, Shizhou Yang, Yuanhe Wang, Jie Luo, Xiaojun Zhu

**Affiliations:** a Department of Gynecology, Women’s Hospital, Zhejiang University School of Medicine, Hangzhou, China; b Department of Obstetrics, Women’s Hospital, Zhejiang University School of Medicine, Hangzhou, China.

**Keywords:** case report, clinicopathologic features, uterine inflammatory myofibroblastic tumor (UIMT)

## Abstract

**Rationale:**

Inflammatory myofibroblastic tumor (IMT) is a rare soft tissue neoplasm with low malignant potential. These patients present with a certain probability of malignant potential. The management of IMT has not been standardized, especially for the patients with fertility needs.

**Patient concerns and Diagnoses:**

Thirteen patients with IMT who attended in the Department of Gynecology, Women’s Hospital, Zhejiang University School of Medicine were enrolled between 2019 and 2023. The data of the clinical and pathological features was analyzed.

**Interventions and Outcomes:**

The mean age of the patients was 45.31 ± 12.80 years. Seven of the 13 cases (53.85%) had abnormal uterine bleeding. Five of them (38.46%) had a rapidly growing mass, and 2 of them (15.38%) had no obvious symptoms. With regard to surgical strategies, 7 patients underwent hysterectomy, and 6 patients underwent mass resection only. All the patients were alive with no evidence of disease at an average of 9.58 months of follow-up. One of them gave birth to a full-term male infant at 40 weeks of gestation after hysteroscopic resection, without recurrence.

**Lessons:**

Uterine inflammatory myofibroblastic tumor can be diagnosed by the anaplastic lymphoma kinase overexpression. Complete excision under hysteroscopy or laparoscopy seems to be effective and safe. Because there is a certain risk of recurrence and metastasis, conservative surgery should be performed carefully to preserve fertility in patients who can undergo close follow-up.

## 1. Introduction

Inflammatory myofibroblastic tumors (IMTs) are low-grade malignant mesenchymal tumors consisting of fibroblastic myofibroblastic cells with inflammatory infiltrates.^[1]^ It occurs in many parts of the body, including the lungs, soft tissue, peritoneum, and bladder. IMT in the female genital tract is rare, with the uterus being the most common location.^[2]^ This phenomenon was first reported by Gilks (1987).^[3]^

The clinical manifestations of uterine IMT (UIMT) are usually nonspecific and usually present as abnormal uterine bleeding, pelvic mass, pelvic pain, arthralgia, low-grade fever, and incidental findings. Histologically, IMT is purely fascicular or myxoid, or shows a predominance of 1 pattern.^[4]^ Cytologically, IMT is characterized by spindle-to-epithelioid cells with myofibroblastic differentiation, some degree of smooth muscle differentiation, and myxoid stroma and is usually associated with brisk lymphoplasmacytic infiltrates.^[5]^ Therefore, misclassification as a uterine leiomyoma or endometrial stromal tumor may occur because of overlapping morphological features.^[6,7]^

Approximately 50% of patients with UIMT have anaplastic lymphoma kinase (ALK) gene rearrangement on chromosome 2p23 with subsequent overexpression of ALK protein,^[8]^ making ALK a specific diagnostic marker for UIMT.^[9]^ Molecular characterization and the application of immunohistochemistry (IHC) and next-generation sequencing have improved the clinical recognition and accurate diagnosis of UIMT.^[10]^ Most UIMT cases can be cured by surgery because its clinical behavior is usually indolent, although it has a certain incidence of recurrence, malignancy, metastasis, or even death.^[11]^ Patients with ALK rearrangements can benefit from ALK inhibitors,^[12]^ if they experience recurrence, malignancy, metastasis, or a contraindication to surgery.

UIMT has only been the subject of a few reports, possibly because of the low incidence and low awareness among clinicians. The role of ALK inhibitor therapy in patients with UIMT is gradually being appreciated; therefore, it is crucial to precisely diagnose UIMT.^[13]^ The clinical and pathological features of 13 patients in the gynecological department are presented in this study.

## 2. Materials and methods

All UIMT cases were retrieved from the Department of Gynecology, Women’s Hospital, Zhejiang University School of Medicine, Zhejiang Province, China. Thirteen patients diagnosed with pathologically confirmed UIMT were enrolled in our study. Clinical data were collected from clinician records and telephone follow-up between 2019 and 2023. This study was approved by the Hospital Research Ethics Committee (ethics reference: IRB-20230144-R).

Hematoxylin and eosin-stained tissue sections and IHC images of all cases were obtained from the pathological databases.

IHC studies were performed using the following primary antibodies: estrogen receptor (clone SP1, Thermo Scientific, USA), Desmin (clone MX046, MXB Biotechnologies, China), smooth muscle actin (SMA, clone 1A4, Novus Biologicals, USA), ALK (clone 5A4, Genscript Biotech, USA), Ki-67 (clone MIB-1, Thermo Scientific, USA), and Caldesmon (clone h-CALD, Thermo Scientific, USA). IHC reaction was graded as positive based on nuclear, cytoplasmic, or membrane reactivity for various antibodies.

Statistical analysis was performed using SPSS version 22.0 (SPSS, Chicago, IL). The results are presented as the mean ± SD or frequency (%).

## 3. Results

### 3.1. The clinical features and outcomes

Table 1 shows the clinical features of all cases. The age ranged from 27 to 67 years (mean, 45.31 ± 12.80 years), and the body mass index ranged from 19.92 to 28.44 (mean, 23.11 ± 2.65). Abnormal uterine bleeding occurred in 7 (53.85%) patients, 1 of whom was postmenopausal. A rapidly growing mass was observed in 5 (38.46%) cases, of which 3 (60.00%) were postmenopausal. Patient 9 had a rapidly growing mass and menorrhagia. Two (15.38%) patients had no obvious symptoms, and UIMT was discovered during routine gynecological examination. One case was found to have intrauterine lesions on ultrasound and the other was found to have cervical lesions incidentally on physical examination. Seven patients had masses in the myometrium, 5 in the uterine cavity, and 1 in the cervix. The diameter of the largest mass ranged from 1 cm to 14 cm (mean, 5.35 ± 3.63 cm).

**Table 1:** Clinical and pathological features of 13 patients with uterine inflammatory myofibroblastic tumor.

								Immunohistochemistry					
Case	Age	BMI	Clinical presentation	Location	Size (cm)	Treatment	Duration of follow-up (months)	ALK	ER	Desmin	SMA	Ki-67	Caldesmon
1	54	20.2	Rapidly growing mass after postmenopause	Myometrium	6	Total abdominal hysterectomy with bilateral oothecosalpingectomy	ANED 3 months	P	P	P	P	1%	P
2	46	25.81	Menorrhagia and abnormal uterine bleeding	Uterine cavity	2	Hysteroscopic mass resection	ANED 1 month	P	NP	P	NP	10%	N
3	50	21.48	Cervical lesions	Cervix	3	Surgically excision of the mass	ANED 5 months	NP	NP	P	N	NP	NP
4	38	24.44	Abnormal uterine bleeding	Uterine cavity	1	Hysteroscopic mass resection	ANED 5 months	P	P	P	N	10%	N
5	37	23.31	Intrauterine lesions	Uterine cavity	2.5	Total laparoscopic hysterectomy with bilateral salpingectomy (Hysteroscopic mass resection was performed 2 weeks ago, and postoperative pathology revealed IMT)	ANED 6 months	P	P	P	P	5%	N
6	63	24.22	Abnormal uterine bleeding after postmenopause	Myometrium	5	Total laparoscopic hysterectomy with bilateral oothecosalpingectomy	NA	P	P	P	P	N	NP
7	31	25.71	Rapidly growing mass	Myometrium	6	Total laparoscopic hysterectomy with bilateral salpingectomy (laparoscopic myomectomy was performed 1 month ago, and postoperative pathology revealed IMT)	ANED 13 months	P	P	P	P	5%	NP
8	56	20.94	Rapidly growing mass after postmenopause	Myometrium	9	Total abdominal hysterectomy with bilateral oothecosalpingectomy	ANED 12 months	P	N	N	N	>50%	N
9	52	28.44	Rapidly growing mass and menorrhagia	Myometrium	9	Total abdominal hysterectomy with bilateral oothecosalpingectomy	ANED 21 months	P	P	P	P	15%	NP
10	36	21.1	Abnormal uterine bleeding	Uterine cavity	2	Hysteroscopic mass resection	ANED 21 months	P	NP	P	NP	<10%	N
11	32	20.5	Abnormal uterine bleeding	Myometrium	5	Laparoscopic myomectomy (uterus retained)	ANED 12 months	P	P	P	P	15%	NP
12	27	19.92	Abnormal uterine bleeding	Intrauterine submucous myomatoid mass protruding within the cervix	5	Hysteroscopic mass resection	ANED 13 months	P	P	P	P	P	NP
13	67	24.41	Rapidly growing mass after postmenopause	Myometrium	14	Total abdominal hysterectomy with bilateral oothecosalpingectomy	ANED 3 months	P	N	P	P	10%	P

ALK = anaplastic lymphoma kinase, ANED = alive with no evidence of disease, BMI = body mass index, ER = estrogen receptor, N = negative, NA = not available, NP = not performed, P = positive, SMA = smooth muscle actin.

Regarding surgical strategies, 7 (53.84%) patients underwent hysterectomy (4 underwent total abdominal hysterectomy with bilateral oothecosalpingectomy, 2 underwent total laparoscopic hysterectomy with bilateral salpingectomy, and 1 underwent total laparoscopic hysterectomy with bilateral oothecosalpingectomy), 6 (46.15%) cases received mass resection only (4 underwent hysteroscopic mass resection, 1 underwent laparoscopic myomectomy, and 1 underwent surgical mass excision).

Considering the risk of recurrence and metastases, cases 5 and 7 underwent hysterectomy 2 weeks and 1 month later, respectively, as the postoperative pathology revealed IMT of the first surgery. The remaining patients did not undergo further surgery or therapy.

Twelve (92.31%) patients were followed up, and 1 patient was lost to follow-up. All the patients were alive with no evidence of disease (including recurrences and/or metastases). The follow-up duration ranged from 1 month to 21 months (mean, 9.58 ± 6.82 months). Patient 12 was pregnant 11 months after hysteroscopic mass resection and gave birth to a full-term male infant at 40 weeks of gestation.

### 3.2. Gross and pathological features

**Figure 1.**
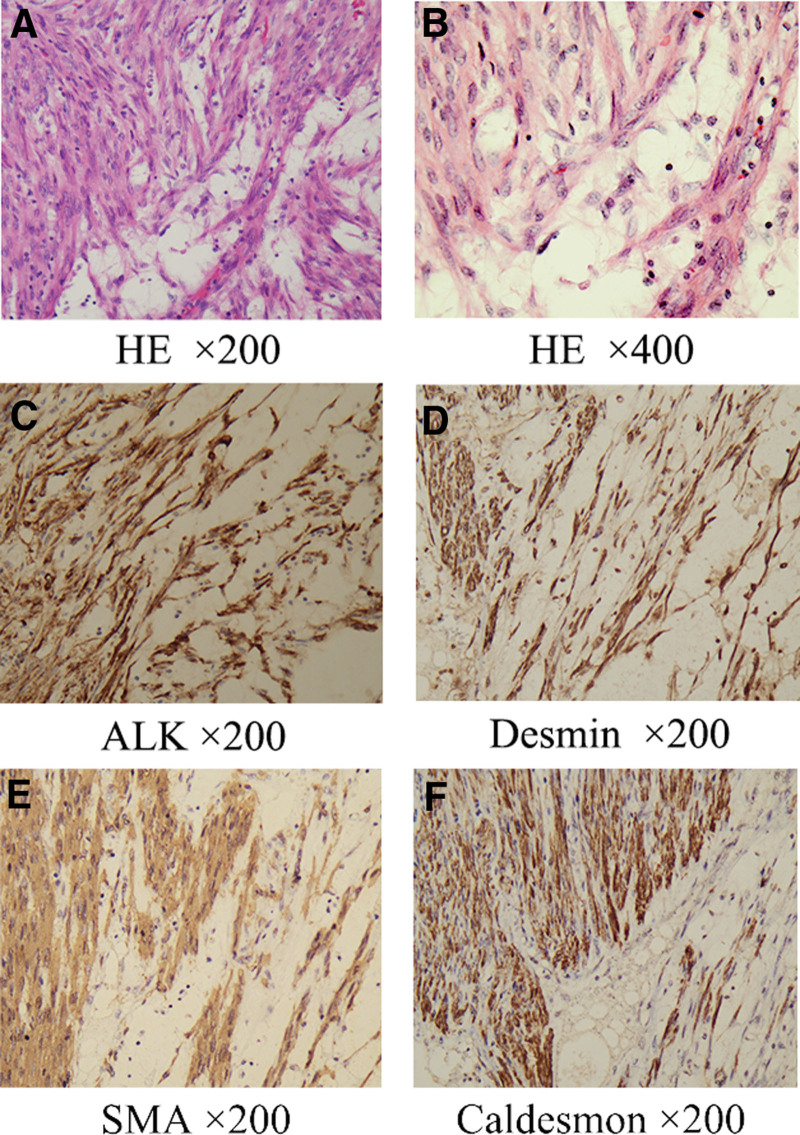
Histopathologic findings of uterine inflammatory myofibroblastic tumor (UIMT). The classic features of UIMT were myxoid hypocellular area with loosely arranged spindle cells and mixed inflammatory infiltrate with HE staining (A and B). And immunohistochemical features of uterine inflammatory myofibroblastic tumor (UIMT). ALK expression in cytoplasm was seen in 100% of cases (C), Desmin expression in cytoplasm was seen in 92.31% of cases (D), SMA expression in cytoplasm was seen in 72.73% of cases (E), and Caldesmon expression in cytoplasm was seen in 28.57% of cases (F). ALK = anaplastic lymphoma kinase, SMA = smooth muscle actin.

It was recorded in the medical records that the majority of the tumors were well-circumscribed on macroscopy. Three patients had a myxoid area, 1 had a necrotic area, 1 had both myxoid and necrotic areas (Fig. 1A), and 1 had a yellow but no whirlpool structure.

All tumors demonstrated the typical histopathological features of UIMT, composed of spindled and epithelioid myofibroblasts in variably myxoid stroma and lymphoplasmic cell infiltration. The myxoid pattern was the most common. It is hypocellular and characterized by a loosely arranged plump to spindle cells in an edematous or myxoid stroma. It was usually accompanied by an irregular network of delicate blood vessels and a prominent mixed inflammatory infiltrate (Fig. 1A and B).

### 3.3. Immunohistochemical features

Results of IHC are summarized in Table 1. Twelve patients (92.31%) underwent IHC for ALK, and ALK overexpression was detected in all cases (100%) (Fig. 1C). Estrogen receptor expression was tested in 10 of 13 (76.92%) cases, and nuclear positivity was observed in 8/10 (80%) cases. Desmin expression was tested in all cases (100%), and cytoplasmic positivity was observed in 12/13 (92.31%) cases (Fig. 1D). SMA expression was tested in 11 of 13 (84.62%) cases, and cytoplasmic positivity was observed in 8/11 (72.73%) cases (Fig. 1E). Caldesmon expression was detected in 7 of 13 (53.85%) cases, and cytoplasmic positivity was observed only in of 2/7 (28.57%) cases (case 1 and case 13) (Fig. 1F).

### 3.4. Blood test features

As shown in Table 2, 1 patient (7.69%) had an elevated white blood cell count, 4 (30.77%) had decreased hemoglobin levels, and 3 (23.08%) had elevated platelet counts. Serum biochemical and hormone test results were negative.

**Table 2:** Auxiliary examination features of 13 patients with uterine inflammatory myofibroblastic tumor.

	Blood test										Imaging	
Case	WBC (×10^9^/L)	RBC (×10^12^/L)	HB (g/L)	PLT (×10^9^/L)	CEA (ng/mL)	AFP (ng/mL)	ca125 (U/mL)	ca153 (U/mL)	ca199 (U/mL)	SCC (ng/mL)	Ultrasound	MRI
1	3	4.38	130	198	0.9	4.1	8.2	7.5	5.4	0.5	Uterine fibroid degeneration	Uterine fibroid degeneration
2	6.3	5.09	96↓	290	1.4	2.7	48.9↑	12.7	11.7	1.4	Submucous myoma	NA
3	5.3	4.09	118	150	NA	NA	58.2↑	NA	8.3	NA	Uterine fibroid	NA
4	6.6	4.4	130	237	NA	NA	NA	NA	NA	NA	Uterine fibroid	NA
5	5.6	4.46	141	333	1.4	3	4.1	7.5	14.8	0.7	mass in uterine cavity	NA
6	8.1	4.77	138	116↓	2	2.7	6.6	15.8	33.2↑	1.7	Uterine fibroid	NA
7	7.1	4.55	139	258	2	1.6	13.6	7	<2	0.7	Uterine fibroid	NA
8	6	3.69	98↓	419↑	0.7	1.2	6	6.1	3.6	0.7	Uterine fibroid	Uterine fibroid degeneration
9	11↑	4.36	128	293	1.1	1.8	7.8	7.9	17	0.5	Uterine fibroid	uterine fibroid
10	6.1	4.17	130	174	NA	NA	NA	NA	NA	NA	Mass in uterine cavity	NA
11	3.9	4.03	65↓	524↑	0.8	7	42.4↑	20.6	37↑	0.5	uterine fibroid	NA
12	6.1	3.95	87↓	290	0.4	1.8	37.6↑	5.7	16.7	0.9	Submucous myoma	Submucous myoma
13	5	4.05	115	281	0.8	5.4	35.8↑	11.1	12.2	1	Uterine fibroid, leiomyosarcoma is not excluded	Uterine fibroid degeneration

**↑ **= above normal, ↓ = below normal, AFP = alpha fetoprotein, CEA = carcinoembryonic antigen, MRI = magnetic resonance imaging, NA = not available.

As for tumor markers, serum carcinoembryonic antigen, alpha fetoprotein, ca153, SCC levels were all normal in the tested 10 cases. Eleven of them (84.62%) had serum carbohydrate antigen 125 (ca125) and ca199 levels. Five of 11 (45.45%) cases had slightly elevated serum ca125 level, and 2 of 11 (18.18%) had slightly elevated serum ca199 level. The serum ca199 level in case 6 decreased to normal on the first day after surgery (Table 2).

### 3.5. Imaging features

Table 2 shows the imaging features of all the cases. All patients underwent ultrasound before surgery, and 2 of them (15.38%) had abnormal signs: 1 was diagnosed with uterine fibroid degeneration, and the other was suspected to have leiomyosarcoma. Case 1 showed a hypoecho mass of 6.58 × 6.92 × 6.36 cm with scattered dark areas inside was found in the anterior wall of the uterus. Blood flow was observed both peripherally and internally (RI, 0.61). Therefore, degeneration of the uterine fibroids was considered.

**Figure 2.**
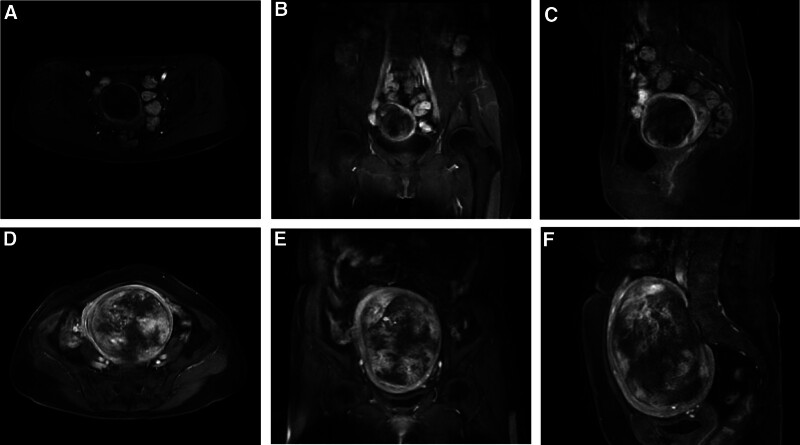
Magnetic resonance imaging (MRI) of uterine inflammatory myofibroblastic tumor (UIMT). A mass with a size of about 6.58 × 6.92 × 6.36 cm (A–C) and 12.2 × 9.8 × 14.7 cm (D–F) occupied in the wall of uterus. Transverse (A and D), coronal (B and E), and sagittal (C and F) sections are observed in the images.

Five (38.46%) patients underwent magnetic resonance imaging (MRI) before surgery, and 3 of 5 (60%) were diagnosed with uterine fibroid degeneration. Case 13 showed a mass with a size of about 12.2 × 9.8 × 14.7 cm occupied the left lateral wall of the uterus. It had a visible edge. A low mixed signal was observed on T1WI, an uneven, slightly high signal on T2WI, and a slightly high signal on DWI. Uneven enhancement was observed after the enhanced scan. Therefore, the mass was considered a myomatous cystic change (Fig. 2A–C (case 1) and Fig. 2D–F (case 13)).

## 4. Discussion

UIMT is an IMT occurring in the uterus. Due to the atypical clinical features, low incidence, and insufficient recognition of UIMT, it has a high chance of being misdiagnosed as a leiomyoma, myxoid leiomyosarcoma, or endometrial stromal tumor, leading to undertreatment or overtreatment. UIMT has a certain risk of recurrence and metastasis; therefore, it is crucial to precisely diagnose it.

We analyzed the 13 cases carefully but found that there was no specificity in clinical manifestations, laboratory examinations, and imaging examinations. Therefore, precise diagnosis is still difficult. However, clinicians should be aware of the possibility of UIMT.

In our study, 11 of 13 (84.62%) patients had symptoms: 7 of 13 (53.84%) patients had abnormal uterine bleeding, and 5 of 13 (38.46%) patients had a rapidly growing mass. Three patients (60.00%) with rapidly growing masses were postmenopausal. Regarding tumor markers, 5 of 11 (45.45%) cases had a slightly higher serum ca125 level. Three (60.00%) of the 5 patients who underwent MRI showed degeneration. Most masses were well-circumscribed but lacked a typical whirlpool structure, 5 (38.46%) had a myxoid and/or necrotic area. Therefore, more attention should be paid to the symptoms of abnormal uterine bleeding and a rapidly growing mass in the uterus, especially after menopause. If the serum ca125 level was slightly higher, MRI indicated degeneration; if the mass had an area of myxoid and/or necrotic area grossly, we should be alert to the possibility of UIMT. ALK is a receptor tyrosine kinase located on chromosome 2p23 that is involved in cellular growth. Approximately 87.5% to 100% of IMT are ALK-positive,^[14]^ but both benign and malignant smooth muscle tumors are ALK-negative, making IHC with ALK specific for IMT. In our study, 12 cases that underwent IHC for ALK were positive, which was consistent with previous studies. So ALK could be used as a specific diagnostic marker for UIMT.^[15,16]^

Clinicians should be familiar with the clinical and histological features of UIMT and make good use of ALK IHC because they are critical for a correct diagnosis. In addition, regular gynecological examinations should be taken seriously, as the 2 cases with no obvious symptoms were diagnosed with UIMT during routine gynecological examinations.

Approximately half of IMT cases harbor a clonal rearrangement of the *ALK* gene.^[17]^ However, many IMT cases with ALK-negative were reported. Appropriate histopathological, immunohistochemical, and sometimes genetic testing are required for correct diagnosis of ALK-negative IMT.^[18]^ Recent advances have revealed that ALK-negative IMTs harbor *ALK* gene rearrangements related to other multiple potentially actionable kinase fusions, such as *RET*, *ROS1*, *ETV6*, *NTRK1*, *NTRK2*, *NTRK3*, and *PDGFRB*,^[19–21]^ giving rise to genome-level research into potential carcinogenic process. Fluorescence in situ hybridization or ribonucleic acid sequencing technologies should be recommended for ALK-negative patients who was suspected for IMT.^[13]^ Also, abnormalities not implicating the *ALK* gene are very rare in uterus tumors, such as *TIMP3-RET*, *TIMP3-ROS1*, and *FN1-ROS1*. One case of *NTRK3* rearrangement has been reported.^[22]^ Maybe IMT with ALK-negative is exactly exist and the mechanism is uncertain. For those ALK-negative cases, WT1 and D2-40 can help its diagnosis.

With the development of IMT recognition, the National Comprehensive Cancer Network (NCCN) Guidelines for Soft Tissue Sarcomas recommend first-generation ALK inhibitors as first-line treatment for IMT with ALK fusions, such as crizotinib, ceritinib, brigatinib, and lorlatinib.^[10,13,23,24]^ Currently, there is no unified standard treatment for UIMT, and surgery, particularly total hysterectomy, is considered the best treatment. In our study, surgeries included total abdominal hysterectomy with bilateral oothecosalpingectomy, total laparoscopic hysterectomy with bilateral salpingectomy or oothecosalpingectomy, laparoscopic myomectomy, hysteroscopic mass resection, and surgical excision of masses. All patients who were available for follow-up were alive with no evidence of disease. One patient became pregnant 11 months after hysteroscopic resection and gave birth to a full-term male infant.

As the incidence of recurrence and metastasis was not very high, mass excision could be performed carefully for patients with childbearing demands who could undergo close follow-up. For patients who are unable to undergo surgical treatment due to complex anatomy, recurrence, or metastasis, but with rearrangement of the ALK gene, targeted therapy with ALK inhibitors has a certain effect.^[10]^ Moreover, patients may derive benefits from second-generation ALK inhibitors after disease progression or intolerance to first-generation inhibitors such as Alectinib.^[12,23]^

A novel clinicopathological risk stratification score assignment can be used to evaluate the prognosis. We scored 1 point each for age > 45 years, size ≥ 5 cm, ≥4 mitotic figures per 10 high-power fields, and infiltrative borders. No tumors with 0 points had an aggressive outcome, whereas 21% of tumors with 1 to 2 points and all tumors with ≥ 3 points had aggressive outcomes. If the clinicopathological risk is high, molecular testing for further risk classification is recommended for improved therapy.^[25]^

In conclusion, UIMT is difficult to diagnose because of its atypical clinical symptoms. Symptoms, such as abnormal uterine bleeding and a rapidly growing mass in the uterus, especially after menopause, should be considered. If there are any abnormalities in tumor markers, imaging features, or appearance of the mass, the possibility of UIMT should be considered to avoid misdiagnosis. ALK is a specific diagnostic marker for IMT. As it carries a certain risk of recurrence and metastasis, total hysterectomy is considered the best treatment. Conservative surgery can be an alternative for patients who desire to preserve fertility and ensure strict follow-up. For patients with ALK gene rearrangement, ALK inhibitors could be an alternative therapy in addition to surgery. However, the conclusion seems a little limited and needs further study which include larger sample size with multi-center study and longer-term follow-up.

## Author contributions

**Conceptualization:** Xiaoming Pan, Jian Zou, Jie Luo.

**Data curation:** Xiaoming Pan, Shizhou Yang.

**Funding acquisition:** Shizhou Yang, Yuanhe Wang.

**Supervision:** Jie Luo, Xiaojun Zhu

**Writing – original draft:** Xiaoming Pan.

**Writing – review & editing:** Jian Zou, Yuanhe Wang.
